# The dltC gene contributes to polyhexamethylene biguanide resistance in *Staphylococcus aureus*

**DOI:** 10.3389/fmicb.2025.1681222

**Published:** 2025-11-13

**Authors:** Chenyang Guo, Congcong Wang, Qihui Chen, Sophia Hao Zheng, Fengji Zhang, Jiayu Yan, Haoyang Brady Long, Jing Luo, Xiaoyan Xuan, Peng Wang, Huaixin Zheng

**Affiliations:** 1 Department of Microbiology and Immunology, School of Basic Medical Sciences, Zhengzhou University, Zhengzhou, China; 2 Keystone Academy, Beijing, China; 3 Department of Animal Biology, College of Agricultural and Environmental Sciences, University of California, Davis, Davis, CA, United States; 4 Nanjing Foreign Language School, Nanjing, China

**Keywords:** *Staphylococcus aureus*, PHMB, bacterial resistance, dlt operon, hydrophobicity

## Abstract

As the efficacy of conventional antibiotics continues to decline due to antibiotic resistance, there is an urgent need for alternative antimicrobial strategies. Polyhexamethylene biguanide (PHMB), a cationic polymer with broad-spectrum antimicrobial activity and low toxicity, has been extensively used in medical and personal care applications. Although no definitive cases of bacterial resistance to PHMB have been reported, resistance to other cationic agents suggests the potential resistance to PHMB. In this study, *Staphylococcus aureus* was cultivated in the presence of a sublethal concentration of PHMB for 30 days, during which the organism developed inheritable resistance. A quantitative proteomics study identified differential expression of the DltC protein, which is associated with cell wall biosynthesis. Our findings revealed structural and chemical alterations in the bacterial cell wall, resulting in a surface with increased hydrophobicity, which leads to PHMB resistance. Furthermore, the adaptive PHMB-resistant strains exhibited elevated sensitivity to the hydrophobic antibiotic chloramphenicol and enhanced resistance to the hydrophilic antibiotics gentamicin and kanamycin, consistent with the resistance mechanism uncovered in this study. These results provide new insights into potential resistance mechanisms against PHMB and offer a foundation for its rational use and future antimicrobial development.

## 1 Introduction

*Staphylococcus aureus* (*S. aureus*) is a common bacterial pathogen first discovered by Alexander Ogston in 1880 (Guo et al., 2020). Clinically significant, it is carried in the nasal cavity of approximately 20–40% of the population (Wertheim et al., 2005; Becker et al., 2017) and can cause infections ranging from mild skin and soft tissue infections (SSTIs) to severe invasive infections, such as pneumonia, bacteremia, infective endocarditis, and osteomyelitis (Lowy, 1998; Humphreys, 2012; Klevens et al., 2007; Rasigade et al., 2014). Additionally, *S. aureus* frequently contaminates medical implants, becoming a major source of nosocomial infections (Tong et al., 2015). Over the past few decades, the incidence of SSTIs has steadily increased globally, with hospitalizations associated with *S. aureus* rising significantly in countries such as the United States, the United Kingdom, and Australia (Hersh et al., 2008; Vaska et al., 2012; Hayward et al., 2008).

The emergence of methicillin-resistant *Staphylococcus aureus* (MRSA) has further complicated the treatment of SSTIs (Mediavilla et al., 2012; Lakhundi and Zhang, 2018). In China, approximately 60% of clinical *S. aureus* isolates are MRSA, with morbidity and mortality rates significantly higher than those of methicillin-sensitive *Staphylococcus aureus* (MSSA) (Xiao et al., 2011; Ippolito et al., 2010). According to data from the U. S. Centers for Disease Control and Prevention (CDC), the mortality rate associated with MRSA infections has surpassed that of AIDS and Parkinson’s disease (Lessa et al., 2012). With the widespread use of antibiotics, the problem of antibiotic resistance in MRSA has become increasingly severe (Chambers and Deleo, 2009; Kuroda et al., 2001). MRSA exhibits resistance to multiple classes of antibiotics, including *β*-lactams, tetracyclines, and aminoglycosides (Jensen and Lyon, 2009). The prevalence of MRSA not only complicates the treatment of *S. aureus* infections but also imposes a significant burden on public health (McCaig et al., 2006). Moreover, community-associated MRSA strains often exhibit higher virulence, such as the production of Panton-Valentine leukocidin (PVL), further exacerbating the severity of infections (Francis et al., 2005; Herold et al., 1998).

In the face of the growing issue of antibiotic resistance, there is an urgent need to develop and utilize novel antimicrobial agents. Polyhexamethylene biguanide (PHMB) is a cationic polymer with broad-spectrum antibacterial activity and has been widely used in the medical and personal care fields (Allen et al., 2004; Mori et al., 2007; Lucas et al., 2009; Hubner and Kramer, 2010; Alves et al., 2021; Asiedu-Gyekye et al., 2015). Studies have shown that PHMB promotes wound healing, reduces bacterial load, and effectively kills *S. aureus* and *Klebsiella pneumoniae* (To et al., 2016; Yim et al., 2023). The cationic structure of PHMB allows it to interact with the negatively charged bacterial cell membrane, thereby disrupting the membrane and causing bacterial death (Scientific Committee on Consumer and Bernauer, 2015; Ntow-Boahene et al., 2023). Additionally, PHMB is able to enter cells, bind to DNA, and inhibit bacterial replication (Chindera et al., 2016; Sowlati-Hashjin et al., 2020).

Although studies have shown that the use of antimicrobial agents in personal care products has minimal impact on microbial resistance, associated risks still exist (Gilbert and McBain, 2003). PHMB exhibits good antibacterial activity, but its long-term use may pose a risk of bacterial resistance. While no formal reports of PHMB resistance have been made (Wessels and Ingmer, 2013), studies have observed bacterial resistance to other cationic disinfectants, such as chlorhexidine (CHX) (Huang et al., 2022; Stickler, 2002). This study investigated whether prolonged exposure to sublethal dose of PHMB could lead to the development of resistance in *S. aureus*, and applied techniques such as proteomics to analyze the mechanisms of resistance. The results elicited a novel mechanism of antimicrobial resistance and provided insights for prevention and control of antimicrobial resistance.

## 2 Materials and methods

### 2.1 Bacterial strains and culture methods

This study strictly adheres to the “Ethical Review Measures for Human Biomedical Research” published by the National Health and Family Planning Commission of the People’s Republic of China and has obtained informed consent from all participants. The bacterial strains used in this study include Methicillin-resistant *Staphylococcus aureus* (MRSA) ATCC 43300, *Staphylococcus aureus* ATCC 25923, *Escherichia coli* DH5α, and *Staphylococcus aureus* RN4220 (this strain was induced from NCTC 8325 by ultraviolet and chemical methods to form a mutant with a defective restriction endonuclease). All strains were obtained through commercial purchase or laboratory donation.

For culture conditions, the *Escherichia coli* DH5α strain was cultured using LB (Luria-Bertani) medium, while the other *Staphylococcus aureus* strains were cultured in TSB (Tryptic Soy Broth) or B2 broth (Sato'o et al., 2018) to ensure appropriate experimental conditions and data reliability.

### 2.2 Growth curve determination

Bacterial strains were inoculated into fresh TSB medium and incubated overnight at 37 °C with shaking at 220 rpm for 15–16 h. Then, 400 μL of the overnight culture was transferred to 40 mL of fresh TSB medium at a 1:100 dilution for subculturing. The growth curve was monitored using a microplate reader at an optical density of 600 nm (OD_600_), with absorbance measurements taken every hour. Three independent replicates were performed for each experiment.

### 2.3 Minimum inhibitory concentration (MIC) determination of PHMB

A 200 μL aliquot of the overnight bacterial culture was diluted 1:100 into 20 mL of fresh tryptic soy broth (TSB) and incubated at 37 °C with shaking at 220 rpm for 3–4 h until reaching the logarithmic growth phase. Then, 40 μL of this culture was inoculated into 4 mL of TSB containing various concentrations of PHMB and incubated under the same conditions for 24 h. OD_600_ was measured using a microplate reader to determine the MIC of PHMB.

Two types of culture containers were used. Initially, MIC assays and resistant strain screening were conducted in 96-well microtiter plates. For subsequent assays involving dltC overexpression strains, sterile glass tubes were used. Aside from the difference in containers, all other procedures were identical.

As PHMB increases in concentration, it leads to increased solution turbidity, independent of bacterial growth. Therefore, MIC was determined by the change in OD_600_ between 0 h and 24 h. When no change in OD_600_ was observed, the corresponding PHMB concentration was defined as the MIC.

### 2.4 Spot test

Spot tests were performed essentially as described elsewhere (Wood et al., 2018). A series of gradient dilutions were prepared from bacterial cultures in the logarithmic phase, diluted up to 1:10^7^. Using a multichannel pipette, 5 μL of each diluted bacterial suspension was spotted onto TSB agar plates containing different concentrations of PHMB. The plates were then incubated overnight at 37 °C in a constant-temperature incubator to observe colony growth.

### 2.5 Transmission electron microscopy observation

TEM observation was performed as previously described (Li et al., 2021). Briefly, 3 mL of overnight culture was centrifuged, and the bacterial pellet was washed three times with PBS. The pellet was fixed in 2.5% glutaraldehyde at 4 °C for 12 h, followed by three PBS washes and pre-embedding in 1% agarose. Fixation was continued with 1% osmium tetroxide in 0.1 M PBS at room temperature for 2 h. After PBS washes, samples were dehydrated through an ethanol gradient, embedded in acetone and 812 resin, sectioned, and stained with 2% uranyl acetate and lead citrate. Images were acquired using transmission electron microscope by Servicebio (Wuhan, China).

### 2.6 Proteomics analysis

Proteomic analysis was performed as previously described (Gao et al., 2023) by Gene Create Biolabs Inc. (Wuhan, China). A 250 mL overnight culture was centrifuged, and the pellet was washed three times with PBS. Protein extraction buffer (7 M urea, 2 M thiourea, 4% SDS, 40 mM Tris–HCl pH 8.5, 1 mM PMSF, 2 mM EDTA) was added, and the sample was mixed and incubated on ice for 5 min. DTT was added to 10 mM, followed by sonication on ice for 15 min. After centrifugation at 13,000 × g at 4 °C for 20 min, the supernatant was mixed with four volumes of cold acetone and stored at −20 °C overnight. The resulting protein pellet was collected, air-dried, and dissolved in 8 M urea/100 mM TEAB (pH 8.0). DTT (10 mM) was added for reduction at 56 °C for 30 min, followed by alkylation with 55 mM IAM at room temperature for 30 min in the dark. Protein concentration was determined using the Bradford method, and samples were labeled for mass spectrometry.

### 2.7 Construction of gene knockout and overexpression plasmids

Gene editing was performed according to established protocols for MRSA (Ji, 2020), using the pCasSa plasmid developed by Ji Quanjian’s lab (Chen et al., 2017). The sgRNA spacer sequence targeting dltC was inserted into the pCasSa plasmid using *Bsa*I, yielding pCasSa_dltC_spacer. A ~ 1 kb upstream and downstream homologous arm of dltC was amplified and fused via overlap extension PCR as a repair template, which was inserted into pCasSa_dltC_spacer using *Xho*I and *Xba*I to generate the knockout plasmid pCasSa_dltC.

For overexpression, the dltC gene and its native promoter were cloned into the multiple cloning site of the pLI50 vector using *EcoR*I and *Xba*I, generating the plasmid pLI50_dltC for dltC overexpression (Table 1).

**Table 1:** Primers used in this work.

Primer name	Sequence(5′ → 3′)
Upstream 5’F-1	TTTGAGATCTGTCCATACCCATGGTCTAGACGTCGTATGGCAAGTTTTATTGA
Upstream 3’R	TTAAATTCTCCTTTATTATATAAGTTTACCTGAGAAGATTAAAAAGCC
Downstream 5’F	TAAACTTATATAATAAAGGAGAATTTAAATGAAATTAAAACCTTTTTT
Downstream 3’R − 1	AAGATACAGGTATATTTTTCTGACTCGAGCCGATGTGTACGGCATCACT
promoter-F-*Xba*I	GTCGACTCTAGAGGATATGTGATGAGTTTATTTGAT
promoter-R	ATTGCACCTCTTAAAGTTCTTAGTAAAAACGC
dltC-F	GAACTTTAAGAGGTGCAATTTGGCTTTTTAATCTTCTCA
dltC-R-EcoRI	TCTTCAAGAATTCGAGAATACCGCTCCACTAATTAA
PCRVerify-F	CGAACGACCTACACCGAACT
PCRVerify-R	CACACATCCAGGTGGTTCAT
dltC-sgRNA-F-1	GAAAGACGTAGAAATTTTTGAAGA
dltC-sgRNA-R-1	AAACTCTTCAAAAATTTCTACGTC
pLI50-insert-F	CTAAAAACCTACAGAAGCTTGCATGCCT
pLI50-AmpR-F	AATGGTTTCTTAGACGTCAGGT
pLI50-AmpR-R	CAAAAAGGATCTTCACCTAGATCC
out-dltC-F	TGCAATGTCTAACGTGGCAT
in-dltC-F	TGATTCTTTCCAAACAGTTGGATT
in-dltC-R	TGGTGTTGCCCACTCATCTC
*gyrA*-F	TCCCTGAATCAACATTACGTCC
*gyrA*-R	CCCTACAACTTCGTCACCTTC

### 2.8 Plasmid electroporation

Electrocompetent cells of *Staphylococcus aureus* RN4220, ATCC 25923, and MRSA ATCC 43300 strains were prepared. The recombinant plasmid pLI50_dltC was electroporated into RN4220 using the BioRad Gene Pulser Xcell™ system with the following parameters: 0.2 cm cuvette, 2.9 kV, 25 μF, and 100 *Ω*. Immediately after electroporation, 1 mL of B2 broth was added, and the cells were incubated at 37 °C with shaking at 100 rpm for 2 h. The suspension was then plated onto TSB agar containing 5 μg/mL chloramphenicol (Cm) and incubated at 37 °C for 2 days. Positive clones were screened by PCR. Verified transformants were cultured in TSB containing 5 μg/mL Cm at 37 °C for 15–16 h. The pLI50_dltC plasmid was extracted from RN4220 (pre-treated with 20 mg/mL lysozyme and 30 U/mL lysostaphin) and subsequently electroporated into MRSA ATCC 43300 and ATCC 25923 competent cells using the same electroporation parameters, adjusting the Cm concentration to 7 μg/mL.

The pCasSa_dltC plasmid was introduced following the same procedure, except that incubation was performed at 30 °C.

### 2.9 Hydrophobicity test

Bacterial surface hydrophobicity was assessed as described by Buchanan et al. (2005), based on the adsorption of bacteria to *n*-hexadecane. An overnight culture (200 μL) was diluted 1:100 into 20 mL fresh TSB and incubated at 37 °C with shaking at 220 rpm for 12 h. After incubation, the bacterial cells were washed three times with PBS and resuspended in PBS. The suspension was adjusted to OD_600_ = 1. Then, 1 mL of this suspension was mixed with 300 μL of *n*-hexadecane, vortexed for 2 min, and left to stand at room temperature for 30 min. The OD_600_ of the lower aqueous phase was measured to assess hydrophobicity using the following formula:

Bacterial hydrophobicity = (OD_600_ before adsorption - OD_600_ after adsorption) / (OD_600_ before adsorption) × 100%.

### 2.10 RNA extraction and reverse transcription quantitative PCR

Total RNA from bacteria was extracted using the Thermo Scientific GeneJET RNA Purification Kit according to the manufacturer’s instructions, with a pre-treatment using 20 mg/mL lysozyme and 30 U/mL lysostaphin. After DNA removal, RNA samples were subjected to cDNA synthesis using Vazyme’s (Nanjing, China) 4 × gDNA wiper Mix and 5 × ChamQ Universal SYBR qPCR Master Mix. The qPCR experiment was conducted using Servicebio’s (Wuhan, China) 2 × Universal Blue SYBR Green qPCR Master Mix and performed on a Thermo (China) QuantStudio 6 Flex fluorescence quantitative PCR system. All PCR reactions were conducted in PCR tubes produced by BBI (Shanghai, China), with the *gyrA* gene used as the internal control. Gene expression levels were calculated using the 2^^(-ΔΔCt)^ method.

### 2.11 Bioinformatics analysis

Bioinformatics analyses in this study were conducted within the RStudio environment using R version 4.2.3. Data preprocessing, statistical analysis, and visualization were performed with relevant bioinformatics packages and tools available in R. The analysis workflow was designed to ensure accuracy and reproducibility of the results.

### 2.12 Statistical analysis

All experiments were conducted with three independent parallel groups to ensure the reliability and reproducibility of the data. Statistical analyses were performed using IBM SPSS Statistics 25 software. Differences between experimental and control groups were assessed using *t*-tests and non-parametric tests. A significance level of *p* < 0.05 was considered statistically significant. Additionally, GraphPad Prism 8.0.2 software was used for data visualization to intuitively display the distribution and differences between groups (Table 1).

## 3 Results

### 3.1 The MRSA strain exhibited resistance after prolonged exposure to PHMB

**Figure 1:**
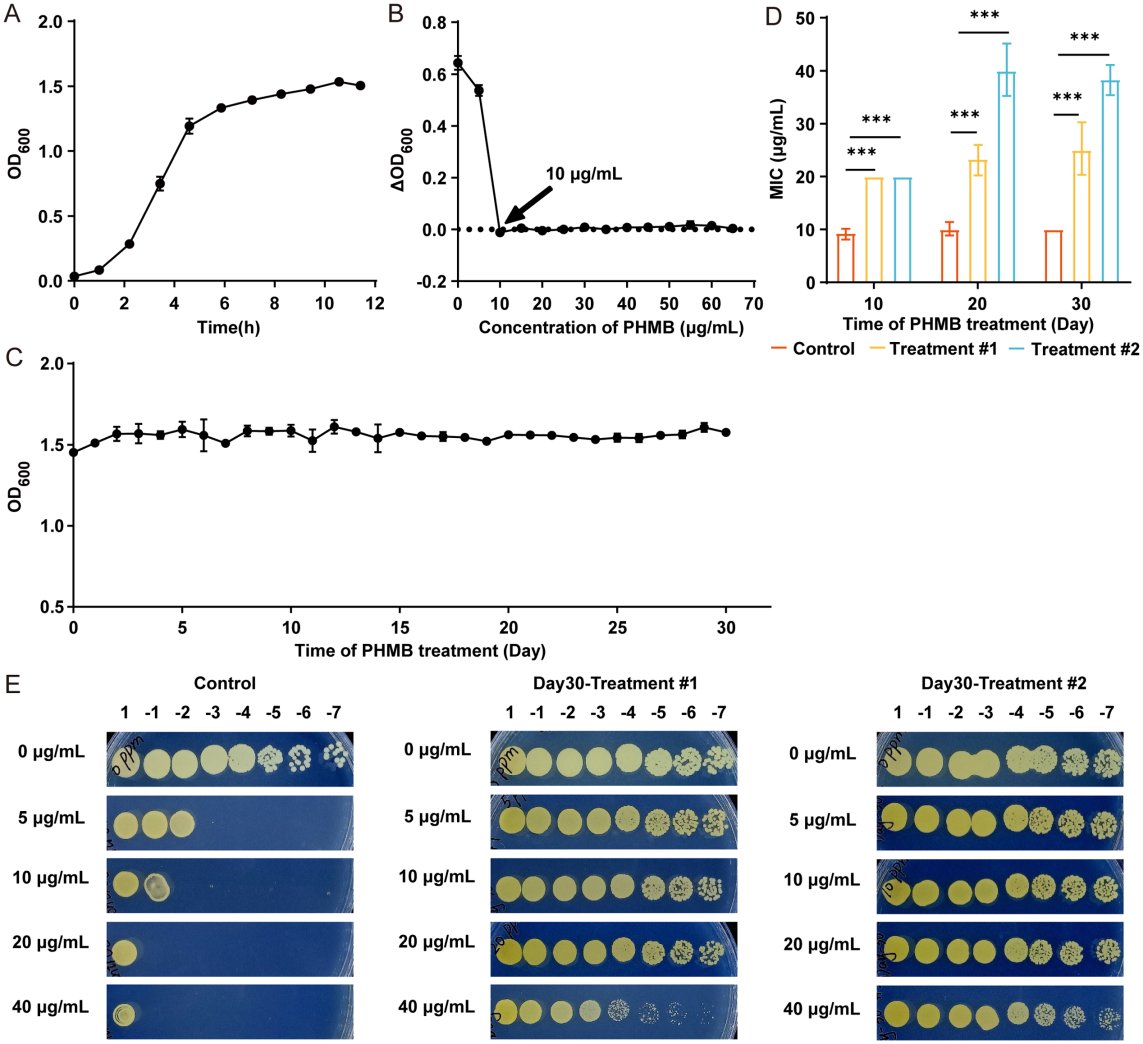
*S. aureus* developed resistance after continuous exposure to 2 μg/mL PHMB. **(A)** Growth curve of MRSA ATCC 43300 based on OD_600_. **(B)** MIC of PHMB determined by OD_600_ changes after 24 h. **(C)** OD_600_ changes during 30 days of continuous PHMB exposure. **(D)** Comparison of MIC values between control and treatment groups on days 10, 20, and 30. **(E)** Resistance assessment of MRSA-PR via spot plating after 1:10^7^ dilution. One control and two treatment groups were analyzed with two technical replicates per condition. Data shown as mean ± SD from ≥3 independent experiments. ***: *p* < 0.001.

The growth curve of the MRSA ATCC 43300 strain (hereafter referred to as MRSA) was first established (Figure 1A), and the MIC of PHMB against MRSA was determined to be 10 μg/mL (Figure 1B). MRSA was continuously passaged for 30 days in TSB containing 2 μg/mL PHMB, maintaining stable growth (OD_600_ ≈ 1.5; Figure 1C). By day 10, the MIC in both treatment groups increased to 20 μg/mL, while the control remained at 10 μg/mL (Supplementary Figure S1A). On day 20, treatment group #1 remained at 20 μg/mL, while group #2 increased to 35 μg/mL (Supplementary Figure S1B). These MIC values persisted through day 30 (Supplementary Figure S1C). Overall, PHMB MIC showed a progressive increase during selection, indicating the emergence of resistant MRSA strains (Figure 1D), further confirmed by spot test (Figure 1E).

For subsequent experiments, MRSA strains with elevated MICs after 30-day exposure were designated as PHMB-resistant MRSA (MRSA-PR). From each of Treatment #1 and Treatment #2, 10 colonies were isolated; four colonies (MRSA-PR #1.2, #1.8, #2.4, #2.9) were randomly selected for further analysis (Supplementary Figure S1D), all showing MICs of 20 μg/mL (Supplementary Figures S1E–I), indicating stable resistance phenotypes.

### 3.2 Physicochemical properties of MRSA-PR

**Figure 2:**
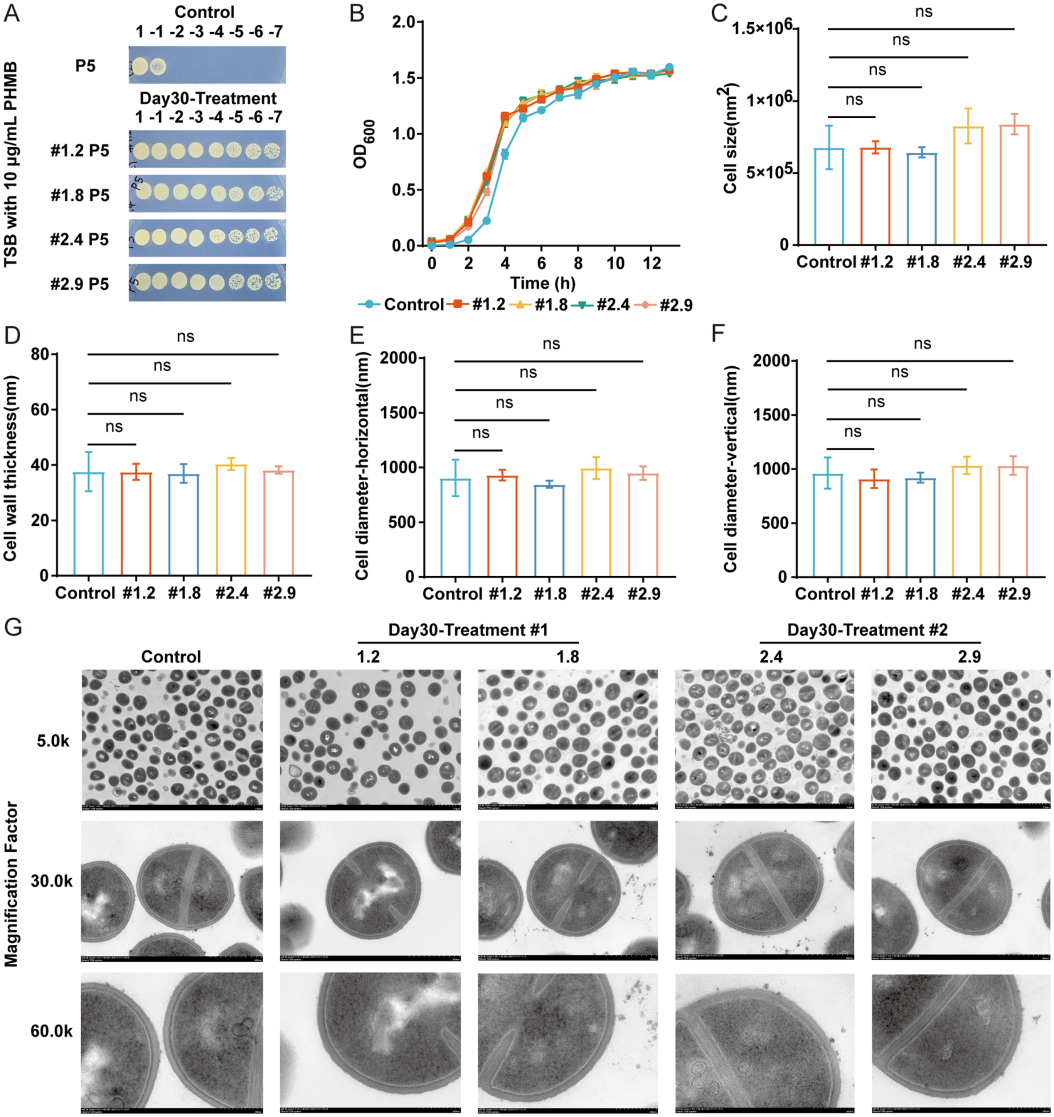
MRSA-PR strains exhibit inheritable feature and show similar growth and cell morphology to the wild-type strain. **(A)** Spot test of MRSA-PR after five passages, using 10-fold serial dilutions plated on TSB agar with 10 μg/mL PHMB. **(B)** Growth curves of MRSA-PR strains (#1.2, #1.8, #2.4, #2.9) and wild-type. **(C–F)** Quantitative comparison of cell morphology: surface area **(C)**, cell wall thickness **(D)**, transverse **(E)** and longitudinal diameter **(F)**. **(G)** TEM images of wild-type and MRSA-PR strains at 5.0 k, 30.0 k, and 60.0 k magnifications. Data shown as mean ± SD from ≥3 experiments. ns: not significant.

To investigate whether the resistance of MRSA-PR is genetically stable, spot test were performed after passaging the four MRSA-PR groups five times in TSB liquid medium without PHMB. The fifth-generation MRSA-PR strains still exhibited strong resistance to PHMB, confirming the inheritable feature of their PHMB resistance (Figure 2A). The growth state, cell morphology, and structural characteristics of MRSA-PR were compared to those of the wild-type strain. Growth curves showed no significant difference between MRSA-PR and wild-type strains (Figure 2B), indicating resistance did not impair growth. TEM analysis revealed no significant differences in cell area, cell wall thickness, transverse cell diameter or longitudinal cell diameter between MRSA-PR and wild-type cells (Figures 2C–G).

### 3.3 Upregulation of DltC protein expression in MRSA-PR

**Figure 3:**
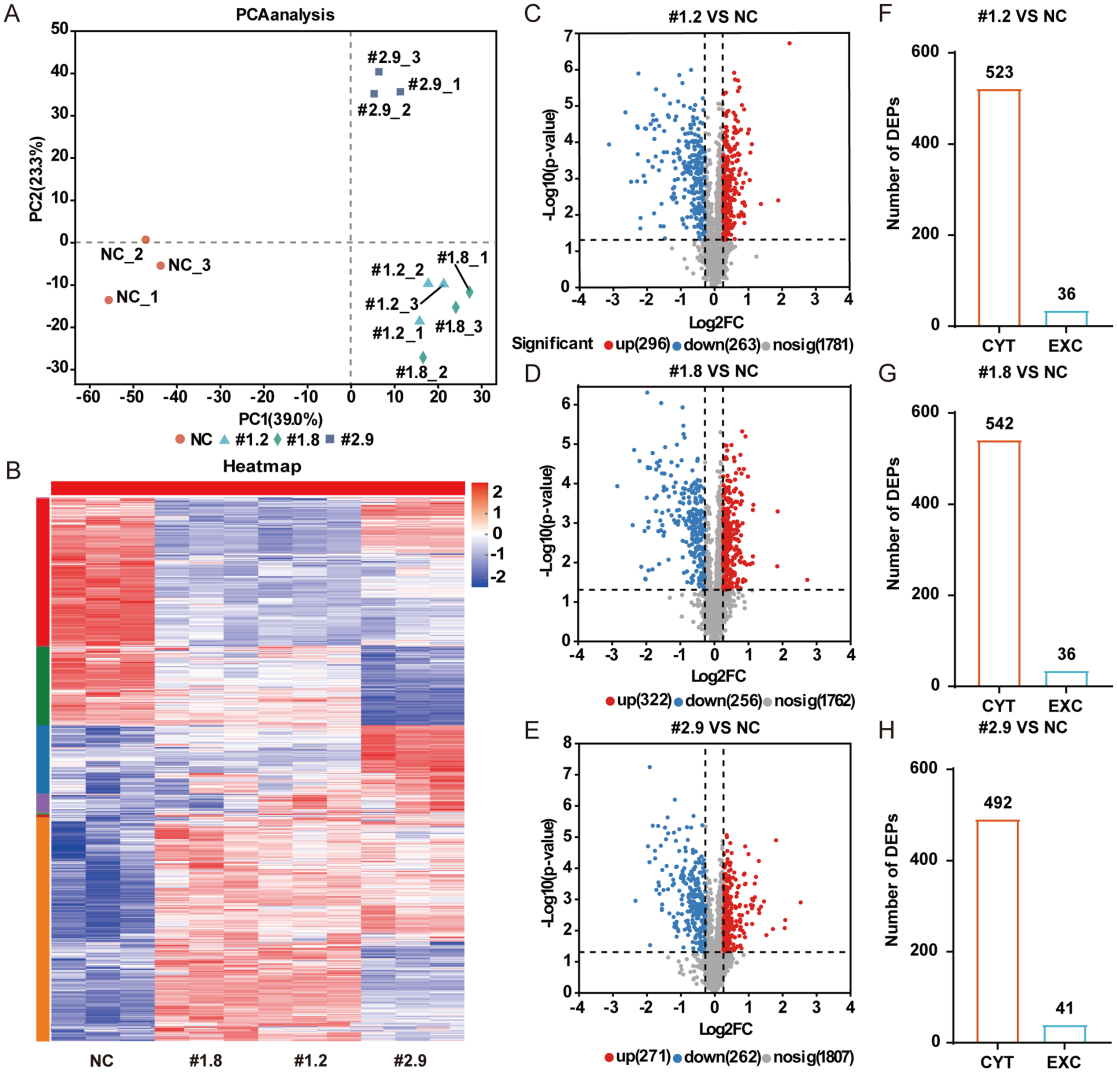
MRSA-PR strains show distinct protein expression profiles compared to the wild-type. **(A)** PCA showing protein expression similarities between MRSA-PR (#1.2, #1.8, #2.9) and wild-type. Each group includes three biological replicates. **(B)** Heatmap of protein abundance across strains. Color intensity reflects relative protein expression levels. **(C–E)** Volcano plots of DEPs: MRSA-PR vs. wild-type. Red: upregulated; blue: downregulated; gray: not significant. **(F–H)** Subcellular localization of DEPs in MRSA-PR vs. wild-type. CYT: cytoplasmic; EXC: extracellular.

To explore the differences in protein expression between the resistant strain MRSA-PR and its parental MRSA strain, proteomic analysis was conducted. Proteomic analysis revealed notable differences in protein expression between MRSA-PR and wild-type strains (Figure 3A). MRSA-PR #1.2 and #1.8 showed similar expression profiles (Figure 3B). Using a threshold of |Log2FC| > 0.3 and *p* < 0.05, MRSA-PR #1.2 had 296 upregulated and 263 downregulated proteins (Figure 3C), #1.8 had 322 upregulated and 256 downregulated (Figure 3D), and #2.9 had 271 upregulated and 262 downregulated (Figure 3E). Subcellular localization showed most DEPs were intracellular, with an approximate 10:1 ratio (Figures 3F–H).

**Figure 4:**
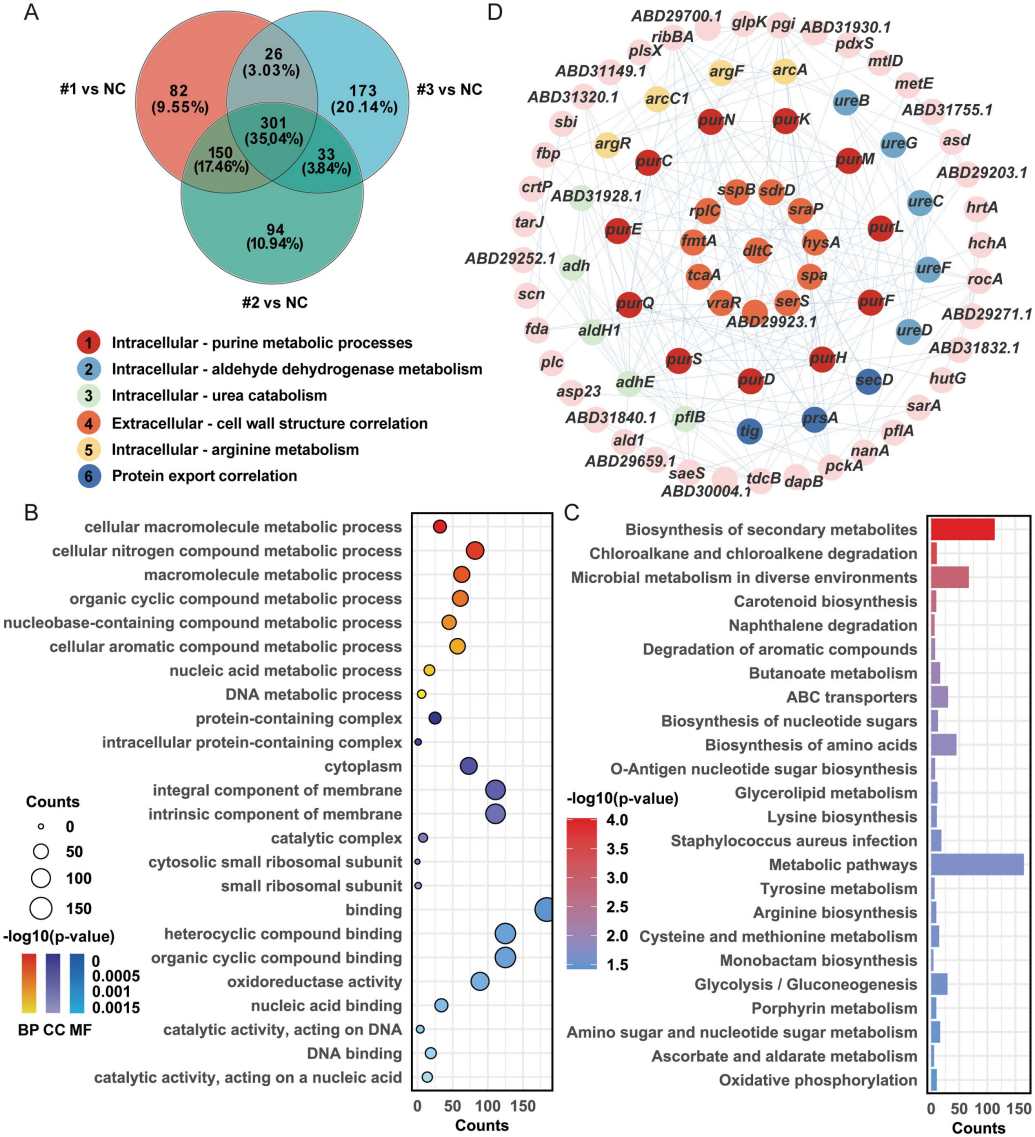
The D-alanyl-D-alanine carrier protein (DltC) is upregulated in MRSA-PR strains. **(A)** Venn diagram of shared DEPs among MRSA-PR strains. A total of 301 DEPs were common to all. **(B)** GO enrichment analysis of shared DEPs. Top 8 terms per category —biological process (BP), cellular component (CC), and molecular function (MF)—were selected based on -log10(*p*-value). **(C)** KEGG pathway enrichment. Top 24 pathways were selected according to -log10(*p*-value). **(D)** PPI network of shared DEPs. Top 6 clusters identified by MCODE, color-coded by cluster.

Additionally, GO and KEGG enrichment analyses revealed subtle functional differences among strains, particularly between MRSA-PR #2.9 and the other two (Supplementary Figures S2A–F). Intersection analysis identified 301 shared DEPs (Figure 4A), which underwent further GO, KEGG, and PPI network analysis (Figures 4B–D). Based on these analyses and supporting background research, we identified a key protein — D-alanine–carrier protein (DltC) D-alanine–carrier protein ligase (DltC) — which was significantly upregulated in the MRSA-PR strains.

**Figure 5:**
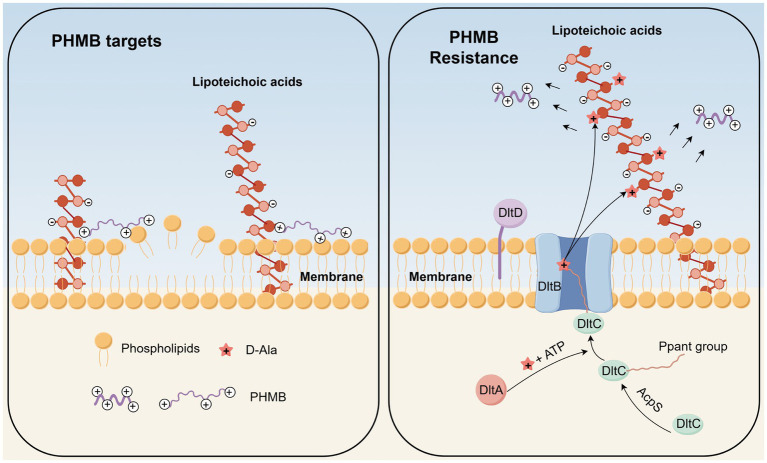
Schematic representation of the D-alanylation of lipoteichoic acid mediated by the dltC gene. DltA exhibits catalytic activity. DltB is a member of the membrane-bound O-acyltransferase (MBOAT) superfamily, consisting of a ring-like structure formed by 11 peripheral transmembrane helices. DltC is an acyl carrier protein. The functions of DltD remain unclear. DltC, as an acyl carrier protein, is first modified at its serine 35 (Ser35) residue by the attachment of a 4′-phosphopantetheine (Ppant) group, catalyzed by acyl carrier protein synthase (AcpS). The Ppant group, derived from the conjugation of pantothenic acid and cysteine, enables DltC to be loaded with D-alanine via ATP consumption, catalyzed by DltA. Upon interaction with DltB, the D-alanyl group is subsequently transferred and attached to lipoteichoic acid (LTA). (This Figure was created using Figdraw).

DltC, encoded by the dltC gene within the dlt operon (dltA-D), plays a key role in lipoteichoic acid (LTA) D-alanylation, affecting cell wall structure and surface charge (Santa Maria et al., 2014; Arnaud et al., 2004; Peschel et al., 1999; Peschel et al., 2000; Heaton and Neuhaus, 1994). DltC, as an acyl carrier protein, is post-translationally modified at Ser35 with phosphopantetheine (Ppant) by AcpS (Ma et al., 2018), enabling it to bind D-alanine via ATP hydrolysis (catalyzed by DltA) and transfer it to LTA through interaction with DltB (Figure 5). D-alanylation reduces LTA’s negative charge, altering cell surface hydrophobicity.

To investigate the potential regulatory mechanism underlying dltC upregulation, whole-genome resequencing was performed to compare the genomic sequences of MRSA-PR strains and the wild-type strain, with a focus on dltC and its associated regulatory regions (data not shown). No mutations were identified in the dltC coding region or its proximal regulatory sequences.

### 3.4 Increased cell surface hydrophobicity of MRSA-PR strains

**Figure 6:**
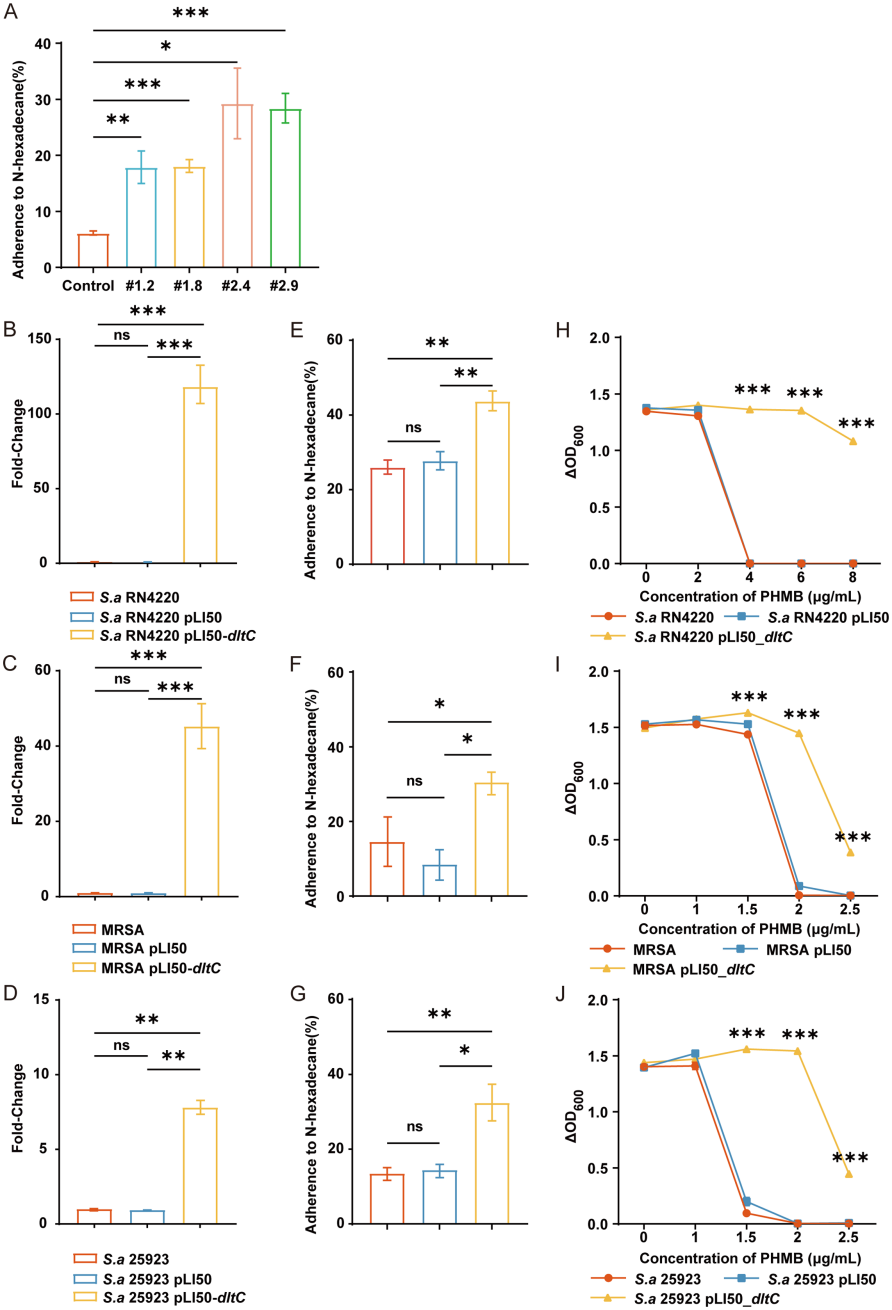
Overexpression of dltC significantly enhances PHMB resistance in *S. aureus.* **(A–C)** Introduction of pLI50_dltC into RN4220, MRSA ATCC 43300, and ATCC 25923., and relative quantification of gene expression was performed using the comparative Ct method (2^^(-ΔΔCt)^). **(D–F)** PHMB susceptibility assessed by OD_600_ changes among dltC-overexpressing strains, wild-type, and empty vector controls. **(G–I)** Bacterial surface hydrophobicity measured in the three backgrounds. **(J)** Hydrophobicity comparison between MRSA-PR and wild-type. Hydrophobicity was measured based on the adherence of bacteria to n-hexadecane. Data shown as mean ± SD from ≥3 experiments. ns: not significant; *: *p* < 0.05; **: *p* < 0.01; ***: *p* < 0.001.

To further investigate the resistance mechanisms of MRSA-PR strains, we evaluated changes in bacterial surface hydrophobicity. MRSA-PR strains displayed significantly increased hydrophobicity compared to the wild-type strain, especially MRSA-PR #2.4 and #2.9 (Figure 6A), which correlated with their higher PHMB resistance. Similarly, overexpression of dltC in RN4220, ATCC 25923, and MRSA strains (Figures 6B–D) led to increased surface hydrophobicity (Figures 6E–G).

Overexpression of dltC also enhanced PHMB resistance. In RN4220, the dltC-overexpressing strain grew at 8 μg/mL PHMB, whereas wild-type and vector controls were inhibited at 4 μg/mL (Figure 6H). Similar results were observed in MRSA and ATCC 25923 backgrounds (Figures 6I,J).

### 3.5 Changes in the sensitivity of MRSA-PR to other antimicrobial agents

**Figure 7:**
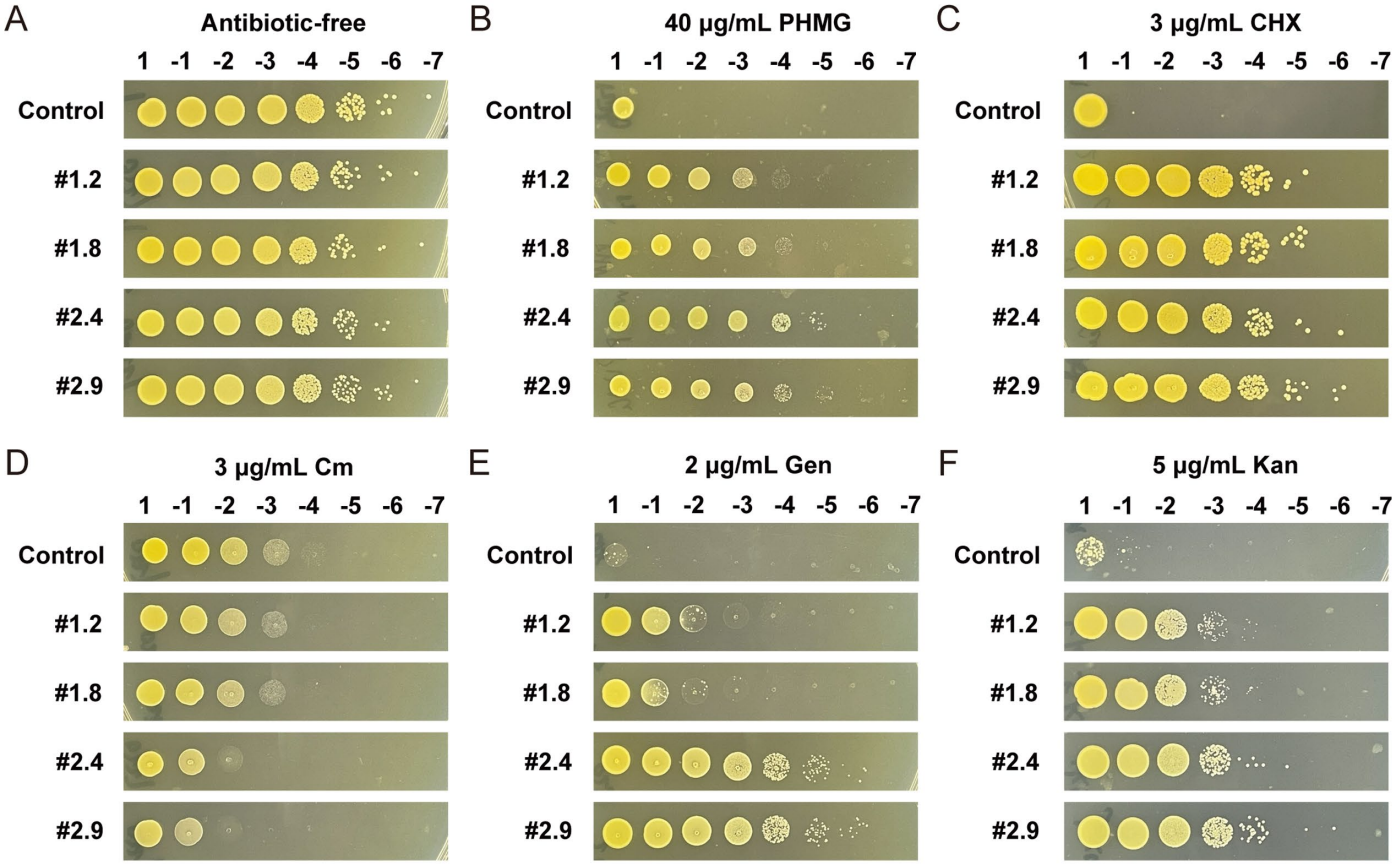
MRSA-PR strains show reduced susceptibility to PHMG and CHX, increased sensitivity to Cm, and enhanced resistance to Gen and Kan. **(A)** The wild-type strain and four MRSA-PR strains were diluted to 1:10^7^ and spotted onto solid agar plates without antimicrobial agents, with two technical replicates per condition. **(B–F)** Susceptibility of MRSA-PR strains was assessed on agar plates containing PHMG **(B)**, CHX **(C)**, Cm **(D)**, Gen **(E)**, and Kan **(F),** using the same dilution and spotting procedure. Two technical replicates per condition.

To investigate whether the MRSA-PR strains exhibit similar resistance changes to other antimicrobial agents, MRSA-PR strains were tested against polyhexamethylene guanidine (PHMG), chlorhexidine (CHX), and antibiotics including chloramphenicol (Cm), kanamycin (Kan), and gentamicin (Gen) by spots test. Without antimicrobials, all strains showed good growth (Figure 7A). At 40 μg/mL PHMG, MRSA-PR strains grew at 10^3^–10^4^ dilutions, while the control only grew undiluted (Figure 7B). At 3 μg/mL CHX, the control again grew only undiluted, while MRSA-PR #1.2 and #1.8 grew at 10^5^ dilutions, and #2.4 and #2.9 at 10^6^ (Figure 7C).

MRSA-PR strains also exhibited altered responses to antibiotics. They were more sensitive to hydrophobic antibiotics (e.g., Cm) but more resistant to hydrophilic ones (e.g., Gen, Kan). At 3 μg/mL Cm, the control grew at a 10^4^ dilution, while MRSA-PR strains only grew at 10^2^–10^3^ (Figure 7D). In contrast, at 2 μg/mL Gen and 5 μg/mL Kan, MRSA-PR strains showed higher resistance than the control, with #2.4 and #2.9 being the most resistant (Figures 7E,F).

## 4 Discussion

The emergence of antimicrobial resistance is a global concern, particularly in clinical settings where resistant strains complicate treatment. *Staphylococcus aureus*, a common pathogen, is notorious for rapidly acquiring resistance. PHMB has demonstrated broad-spectrum bactericidal activity, even at concentrations below 10 μg/mL. Previous research has examined the interactions between PHMB and different membrane types, showing that PHMB rapidly binds to negatively charged membranes (Ikeda et al., 1983; Kuroki et al., 2019), primarily by adsorbing onto the surface of phospholipid bilayers (Horner et al., 2015). Moreover, a recent study proposed that PHMB may enter both bacterial and mammalian cells and selectively condense bacterial chromosomes (Chindera et al., 2016; Allen et al., 2006), this phenomenon not fully explained by membrane interactions alone (Ikeda et al., 1983; Broxton et al., 1984a; Broxton et al., 1984b). According to this new model, PHMB polymer chains can penetrate mammalian cell membranes but not the membrane-bound nucleus where genetic material is stored. In contrast, bacteria—being prokaryotes—lack membrane-enclosed organelles and nuclei, allowing PHMB to potentially interact directly with bacterial chromosomes upon cell entry (Sowlati-Hashjin et al., 2020). Although PHMB, as a novel cationic biocide, exhibits significant efficacy against *S. aureus* and other pathogens, the potential emergence of resistance warrants close attention.

To assess PHMB resistance potential in *S. aureus*, we used the MRSA ATCC 43300 strain. Upon prolonged exposure to sublethal PHMB concentrations, resistant variants with stably elevated MICs were obtained. These findings support earlier reports of acquired biocide resistance, such as chlorhexidine resistance in *Streptococcus mutans* (Verspecht et al., 2019).

This study further explored the growth characteristics of the resistant strains. Growth curves of the resistant and wild-type strain were analyzed to determine possible differences in growth patterns. The results showed no significant difference in growth behavior, which contrasts with earlier reports suggesting that resistance acquisition often correlates with a reduced growth rate in bacteria (Theophel et al., 2014). This discrepancy may indicate that PHMB resistance in MRSA does not notably impair growth or metabolic activity. Additionally, TEM imaging also showed no marked morphological changes, differing from prior reports where resistant strains developed thicker cell walls (Mavri and Smole Mozina, 2013). These discrepancies may stem from species-specific structural and resistance mechanisms.

Proteomic analysis identified dltC—a gene encoding the D-alanine–carrier protein within the dlt operon—as significantly upregulated in resistant strains. DltC plays a key role in the D-alanylation of lipoteichoic acid (LTA), a process that reduces the net negative charge of the bacterial cell surface and increases hydrophobicity. This change may hinder PHMB’s binding to the membrane, diminishing its antibacterial effect. To validate this, we constructed dltC overexpression strains, which exhibited significantly increased PHMB resistance. Although a knockout strain could not be obtained—likely due to *S. aureus*’s inherent transformation barriers or limited CRISPR/Cas9 adaptability—our findings are consistent with previous reports linking dltC upregulation to chlorhexidine resistance (Huang et al., 2022).

We also assessed the cell surface hydrophobicity of both the resistant strains and the dltC-overexpressing strains, the results demonstrated a marked increase in hydrophobicity compared to the wild-type strain. This observation is consistent with previous studies (Kitagawa et al., 2016; El-Banna et al., 2019), it has been reported that bacteria that develop resistance show elevated surface hydrophobicity, supporting a potential link between hydrophobicity and antimicrobial resistance. The increase in hydrophobicity is likely linked to dlt operon upregulation, as enhanced LTA D-alanylation reduces the negative charge of teichoic acids, indirectly increasing surface hydrophobicity, thereby improving bacterial survival upon PHMB exposure.

In addition, the altered sensitivity of resistant strains to other cationic agents (e.g., PHMG and CHX) suggests that increased surface hydrophobicity may decrease the binding efficiency of cationic agents, thereby contributing to enhanced resistance in the same way.

For antibiotics, variations in bacterial susceptibility may similarly be influenced by changes in cell surface hydrophobicity: Gentamicin and kanamycin, both belonging to the aminoglycoside class, contain multiple amino and hydroxyl groups, making them highly charged and strongly hydrophilic. In contrast, chloramphenicol, with its aromatic ring, dichloroacetyl, and nitro groups, is more hydrophobic than gentamicin and kanamycin. Upregulation of the dltC gene increases the hydrophobicity of the bacterial cell surface, which preferentially hinders the uptake of hydrophilic aminoglycosides, thereby enhancing *Staphylococcus aureus* resistance to these agents, while conversely enhancing sensitivity to chloramphenicol. Notably, MRSA-PR#2.4 and #2.9 exhibited higher hydrophobicity and corresponding trends in antibiotic resistance compared to MRSA-PR#1.2 and #1.8, further validating this hypothesis. PHMB resistance in *S. aureus* may reshape its antimicrobial susceptibility, potentially driving clinically relevant cross-resistance. For infections with PHMB-resistant strains, antibiotic hydrophilicity and cross-resistance should be considered to guide appropriate drug selection and dosing.

Both MRSA-PR and the dltC-overexpressing strains exhibited significantly higher surface hydrophobicity compared to the wild-type strain. This change may reduce PHMB adsorption, thereby contributing to increased resistance. In this study, we also attempted to measure the Zeta potential of the bacterial cell membrane surface (data not shown), however, the results indicated no significant difference in Zeta potential both the MRSA-PR strains, and the wild-type strain. This result may be attributed to the limited impact of dlt-mediated D-alanylation on the overall surface charge, which may not be sufficient to alter the Zeta potential at a detectable level. Moreover, changes in cell surface hydrophobicity do not necessarily correspond to measurable differences in electrostatic potential, as Zeta potential is influenced by a variety of physicochemical parameters including ionic strength, pH, and surface charge distribution.

Based on whole-genome resequencing data, we examined the sequences of the dlt operon and its associated regulatory genes, such as *graR* and *graS*. These analyses revealed no mutations in the coding regions or proximal regulatory sequences of these genes. These findings suggest that the mechanism driving dltC overexpression remains unclear. It is possible that more complex transcriptional or post-transcriptional regulatory processes, rather than direct sequence changes, contribute to the elevated dltC expression observed in resistant strains. Further studies are needed to elucidate the regulatory pathways involved.

This study has several limitations. Although dltC was significantly up-regulated in the resistant strain and associated with increased surface hydrophobicity and PHMB tolerance, other members of the dlt operon, such as dltA and dltD, showed no significant changes in our proteomic analysis, which may reflect post-transcriptional regulation or limited sensitivity of the proteomic approach. We did not directly quantify D-alanylation of teichoic acids, and genetic validation through knockout experiments was not performed; future studies will focus on constructing dltC deletion mutants to directly confirm its role in PHMB resistance. It should also be noted that the level of dltC overexpression in our experiments may not fully replicate the endogenous up-regulation observed in resistant strains. While our data indicate that dltC plays a major role in modulating surface hydrophobicity and PHMB resistance, surface hydrophobicity is a complex phenotype influenced by multiple genetic and physiological factors, including other cell wall–modifying genes, regulatory pathways affecting teichoic acid composition, and changes in membrane lipid content. Therefore, the increased surface hydrophobicity observed in PHMB-resistant strains likely reflects the combined effects of several factors rather than the sole activity of dltC. These limitations highlight the need for further work to comprehensively elucidate the molecular mechanisms underlying altered surface properties in resistant strains.

Additionally, the upregulation of efflux-related proteins (EcsA, MepB, EmrR, NorB) suggests that active efflux may also contribute to PHMB resistance, which warrants further investigation. While this study focused on *S. aureus*, it remains unclear whether similar mechanisms operate in other Gram-positive species. In Gram-negative bacteria, resistance may involve LPS modification and also warrants further investigation.

In conclusion, this study presents the first systematic investigation of PHMB resistance and its underlying mechanisms in *S. aureus*. Through phenotypic, morphological, genomic, and proteomic analyses, our findings suggest that the upregulation of the dltC gene, leading to increased cell surface hydrophobicity, plays a pivotal role in resistance. These findings provide novel insights into PHMB resistance in *S. aureus* and lay the groundwork for future development of targeted strategies to overcome or inhibit this emerging form of antimicrobial resistance.

## 5 Conclusion

This study successfully selected resistant strains of *Staphylococcus aureus* by repeatedly exposing the bacteria to sublethal concentrations of PHMB. For the first time, it was confirmed that *Staphylococcus aureus* gradually develops resistance under the selective pressure of prolonged PHMB exposure.

The resistance of *Staphylococcus aureus* to PHMB is closely related to the upregulation of the dltC - induced increase in cell surface hydrophobicity.

The upregulation of the dltC expression may be a universal mechanism for *Staphylococcus aureus* resistance to cationic antimicrobial agents, and changes in bacterial hydrophobicity can significantly alter its resistance to antibiotics.

## Data Availability

The mass spectrometry proteomics data have been deposited to the ProteomeXchange Consortium via the PRIDE [1] partner repository with the dataset identifier PXD069566.

## References

[ref1] Allen M. J. Morby A. P. White G. F. (2004). Cooperativity in the binding of the cationic biocide polyhexamethylene biguanide to nucleic acids. Biochem. Biophys. Res. Commun. 318, 397–404. doi: 10.1016/j.bbrc.2004.04.043, PMID: 15120614

[ref2] Allen M. J. White G. F. Morby A. P. (2006). The response of *Escherichia coli* to exposure to the biocide polyhexamethylene biguanide. Microbiology 152, 989–1000. doi: 10.1099/mic.0.28643-0, PMID: 16549663

[ref3] Alves P. J. Barreto R. T. Barrois B. M. Gryson L. G. Meaume S. Monstrey S. J. (2021). Update on the role of antiseptics in the management of chronic wounds with critical colonisation and/or biofilm. Int. Wound J. 18, 342–358. doi: 10.1111/iwj.13537, PMID: 33314723 PMC8244012

[ref4] Arnaud M. Chastanet A. Debarbouille M. (2004). New vector for efficient allelic replacement in naturally nontransformable, low-GC-content, gram-positive bacteria. Appl. Environ. Microbiol. 70, 6887–6891. doi: 10.1128/AEM.70.11.6887-6891.2004, PMID: 15528558 PMC525206

[ref5] Asiedu-Gyekye I. J. Mahmood A. S. Awortwe C. Nyarko A. K. (2015). Toxicological assessment of polyhexamethylene biguanide for water treatment. Interdiscip. Toxicol. 8, 193–202. doi: 10.1515/intox-2015-0029, PMID: 27486381 PMC4961918

[ref6] Becker K. Schaumburg F. Fegeler C. Friedrich A. W. Kock R. (2017). *Staphylococcus aureus* from the German general population is highly diverse. Int. J. Med. Microbiol. 307, 21–27. doi: 10.1016/j.ijmm.2016.11.007, PMID: 28017539

[ref7] Broxton P. Woodcock P. Gilbert P. (1984a). Binding of some polyhexamethylene biguanides to the cell envelope of *Escherichia coli* ATCC 8739. Microbios 41, 15–22, PMID: 6396498

[ref8] Broxton P. Woodcock P. Heatley F. Gilbert P. (1984b). Interaction of some polyhexamethylene biguanides and membrane phospholipids in Escherichia coli. J. Appl. Bacteriol. 57, 115–124. doi: 10.1111/j.1365-2672.1984.tb02363.x, PMID: 6386785

[ref9] Buchanan J. T. Stannard J. A. Lauth X. Ostland V. E. Powell H. C. Westerman M. E. . (2005). *Streptococcus iniae* phosphoglucomutase is a virulence factor and a target for vaccine development. Infect. Immun. 73, 6935–6944. doi: 10.1128/iai.73.10.6935-6944.2005, PMID: 16177373 PMC1230984

[ref10] Chambers H. F. Deleo F. R. (2009). Waves of resistance: Staphylococcus aureus in the antibiotic era. Nat. Rev. Microbiol. 7, 629–641. doi: 10.1038/nrmicro2200, PMID: 19680247 PMC2871281

[ref11] Chen W. Zhang Y. Yeo W. S. Bae T. Ji Q. (2017). Rapid and efficient genome editing in *Staphylococcus aureus* by using an engineered CRISPR/Cas9 system. J. Am. Chem. Soc. 139, 3790–3795. doi: 10.1021/jacs.6b13317, PMID: 28218837

[ref12] Chindera K. Mahato M. Sharma A. K. Horsley H. Kloc-Muniak K. Kamaruzzaman N. F. . (2016). The antimicrobial polymer PHMB enters cells and selectively condenses bacterial chromosomes. Sci. Rep. 6:23121. doi: 10.1038/srep23121, PMID: 26996206 PMC4800398

[ref13] El-Banna T. Abd El-Aziz A. Sonbol F. El-Ekhnawy E. (2019). Adaptation of *Pseudomonas aeruginosa* clinical isolates to benzalkonium chloride retards its growth and enhances biofilm production. Mol. Biol. Rep. 46, 3437–3443. doi: 10.1007/s11033-019-04806-7, PMID: 30972606

[ref14] Francis J. S. Doherty M. C. Lopatin U. Johnston C. P. Sinha G. Ross T. . (2005). Severe community-onset pneumonia in healthy adults caused by methicillin-resistant *Staphylococcus aureus* carrying the Panton-valentine leukocidin genes. Clin. Infect. Dis. 40, 100–107. doi: 10.1086/427148, PMID: 15614698

[ref15] Gao Z. Li C. Sun H. Bian Y. Cui Z. Wang N. . (2023). N(6)-methyladenosine-modified USP13 induces pro-survival autophagy and imatinib resistance via regulating the stabilization of autophagy-related protein 5 in gastrointestinal stromal tumors. Cell Death Differ. 30, 544–559. doi: 10.1038/s41418-022-01107-8, PMID: 36528756 PMC9950061

[ref16] Gilbert P. McBain A. J. (2003). Potential impact of increased use of biocides in consumer products on prevalence of antibiotic resistance. Clin. Microbiol. Rev. 16, 189–208. doi: 10.1128/CMR.16.2.189-208.2003, PMID: 12692093 PMC153147

[ref17] Guo Y. Song G. Sun M. Wang J. Wang Y. (2020). Prevalence and therapies of antibiotic-resistance in *Staphylococcus aureus*. Front. Cell. Infect. Microbiol. 10:107. doi: 10.3389/fcimb.2020.00107, PMID: 32257966 PMC7089872

[ref18] Hayward A. Knott F. Petersen I. Livermore D. M. Duckworth G. Islam A. . (2008). Increasing hospitalizations and general practice prescriptions for community-onset staphylococcal disease, England. Emerg. Infect. Dis. 14, 720–726. doi: 10.3201/eid1405.070153 18439352 PMC2600225

[ref19] Heaton M. P. Neuhaus F. C. (1994). Role of the D-alanyl carrier protein in the biosynthesis of D-alanyl-lipoteichoic acid. J. Bacteriol. 176, 681–690. doi: 10.1128/jb.176.3.681-690.1994, PMID: 8300523 PMC205105

[ref20] Herold B. C. Immergluck L. C. Maranan M. C. Lauderdale D. S. Gaskin R. E. Boyle-Vavra S. . (1998). Community-acquired methicillin-resistant *Staphylococcus aureus* in children with no identified predisposing risk. JAMA 279, 593–598. doi: 10.1001/jama.279.8.593 9486753

[ref21] Hersh A. L. Chambers H. F. Maselli J. H. Gonzales R. (2008). National trends in ambulatory visits and antibiotic prescribing for skin and soft-tissue infections. Arch. Intern. Med. 168, 1585–1591. doi: 10.1001/archinte.168.14.1585 18663172

[ref22] Horner I. J. Kraut N. D. Hurst J. J. Rook A. M. Collado C. M. Atilla-Gokcumen G. E. . (2015). Effects of polyhexamethylene biguanide and polyquaternium-1 on phospholipid bilayer structure and dynamics. J. Phys. Chem. B 119, 10531–10542. doi: 10.1021/acs.jpcb.5b07162, PMID: 26239890

[ref23] Huang S. Wu M. Li Y. Du J. Chen S. Jiang S. . (2022). The dlt operon contributes to the resistance to chlorhexidine in *Streptococcus mutans*. Int. J. Antimicrob. Agents 59:106540. doi: 10.1016/j.ijantimicag.2022.106540 35092806

[ref24] Hubner N. O. Kramer A. (2010). Review on the efficacy, safety and clinical applications of polihexanide, a modern wound antiseptic. Skin Pharmacol. Physiol. 23, 17–27. doi: 10.1159/000318264 20829658

[ref25] Humphreys H. (2012). *Staphylococcus aureus*: the enduring pathogen in surgery. Surgeon 10, 357–360. doi: 10.1016/j.surge.2012.05.003, PMID: 23079115

[ref26] Ikeda T. Tazuke S. Watanabe M. (1983). Interaction of biologically active molecules with phospholipid membranes: I. Fluorescence depolarization studies on the effect of polymeric biocide bearing biguanide groups in the main chain. Biochim. Biophys. Acta 735, 380–386. doi: 10.1016/0005-2736(83)90152-9, PMID: 6639946

[ref27] Ippolito G. Leone S. Lauria F. N. Nicastri E. Wenzel R. P. (2010). Methicillin-resistant *Staphylococcus aureus*: the superbug. Int. J. Infect. Dis. 14, S7–S11. doi: 10.1016/j.ijid.2010.05.003 20851011

[ref28] Jensen S. O. Lyon B. R. (2009). Genetics of antimicrobial resistance in *Staphylococcus aureus*. Future Microbiol. 4, 565–582. doi: 10.2217/fmb.09.30 19492967

[ref29] Ji Y. (2020). Methicillin-resistant *Staphylococcus aureus* (MRSA) protocols-cutting-edge technologies and advancements. 3rd Edn. Berlin: Springer.

[ref30] Kitagawa H. Izutani N. Kitagawa R. Maezono H. Yamaguchi M. Imazato S. (2016). Evolution of resistance to cationic biocides in Streptococcus mutans and *Enterococcus faecalis*. J. Dent. 47, 18–22. doi: 10.1016/j.jdent.2016.02.008, PMID: 26904979

[ref31] Klevens R. M. Morrison M. A. Nadle J. Petit S. Gershman K. Ray S. . (2007). Invasive methicillin-resistant *Staphylococcus aureus* infections in the United States. JAMA 298, 1763–1771. doi: 10.1001/jama.298.15.1763 17940231

[ref32] Kuroda M. Ohta T. Uchiyama I. Baba T. Yuzawa H. Kobayashi I. . (2001). Whole genome sequencing of meticillin-resistant *Staphylococcus aureus*. Lancet 357, 1225–1240. doi: 10.1016/s0140-6736(00)04403-2, PMID: 11418146

[ref33] Kuroki A. Tchoupa A. K. Hartlieb M. Peltier R. Locock K. E. Unnikrishnan M. . (2019). Targeting intracellular, multi-drug resistant *Staphylococcus aureus* with guanidinium polymers by elucidating the structure-activity relationship. Biomaterials 217:119249. doi: 10.1016/j.biomaterials.2019.119249, PMID: 31279102

[ref34] Lakhundi S. Zhang K. (2018). Methicillin-resistant *Staphylococcus aureus*: molecular characterization, evolution, and epidemiology. Clin. Microbiol. Rev. 31:18. doi: 10.1128/CMR.00020-18, PMID: 30209034 PMC6148192

[ref35] Lessa F. C. Mu Y. Ray S. M. Dumyati G. Bulens S. Gorwitz R. J. . (2012). Impact of USA300 methicillin-resistant *Staphylococcus aureus* on clinical outcomes of patients with pneumonia or central line-associated bloodstream infections. Clin. Infect. Dis. 55, 232–241. doi: 10.1093/cid/cis408, PMID: 22523264 PMC12039758

[ref36] Li M. Cheng L. Tang J. Daroch M. (2021). Molecular components of nitrogen fixation gene cluster and associated enzymatic activities of non-Heterocystous thermophilic cyanobacterium Thermoleptolyngbya sp. Life (Basel) 11:640. doi: 10.3390/life11070640, PMID: 34209262 PMC8307165

[ref37] Lowy F. D. (1998). *Staphylococcus aureus* infections. N. Engl. J. Med. 339, 520–532. doi: 10.1056/NEJM199808203390806, PMID: 9709046

[ref38] Lucas A. D. Gordon E. A. Stratmeyer M. E. (2009). Analysis of polyhexamethylene biguanide in multipurpose contact lens solutions. Talanta 80, 1016–1019. doi: 10.1016/j.talanta.2009.07.031, PMID: 19836589

[ref39] Ma D. Wang Z. Merrikh C. N. Lang K. S. Lu P. Li X. . (2018). Crystal structure of a membrane-bound O-acyltransferase. Nature 562, 286–290. doi: 10.1038/s41586-018-0568-2, PMID: 30283133 PMC6529733

[ref40] Mavri A. Smole Mozina S. (2013). Development of antimicrobial resistance in campylobacter jejuni and *Campylobacter coli* adapted to biocides. Int. J. Food Microbiol. 160, 304–312. doi: 10.1016/j.ijfoodmicro.2012.11.006, PMID: 23290239

[ref41] McCaig L. F. McDonald L. C. Mandal S. Jernigan D. B. (2006). *Staphylococcus aureus*-associated skin and soft tissue infections in ambulatory care. Emerg. Infect. Dis. 12, 1715–1723. doi: 10.3201/eid1211.060190, PMID: 17283622 PMC3372331

[ref42] Mediavilla J. R. Chen L. Mathema B. Kreiswirth B. N. (2012). Global epidemiology of community-associated methicillin resistant *Staphylococcus aureus* (CA-MRSA). Curr. Opin. Microbiol. 15, 588–595. doi: 10.1016/j.mib.2012.08.003, PMID: 23044073

[ref43] Mori K. Hayashi Y. Akiba T. Noguchi Y. Yoshida Y. Kai A. . (2007). Effects of hand hygiene on feline calicivirus inactivation and removal as norovirus surrogate treated with antiseptic hand rubbing, wet wipes, and functional water. Kansenshogaku Zasshi 81, 249–255. doi: 10.11150/kansenshogakuzasshi1970.81.249, PMID: 17564112

[ref44] Ntow-Boahene W. Papandronicou I. Miculob J. Good L. (2023). Fungal cell barriers and organelles are disrupted by polyhexamethylene biguanide (PHMB). Sci. Rep. 13:2790. doi: 10.1038/s41598-023-29756-w, PMID: 36797386 PMC9935507

[ref45] Peschel A. Otto M. Jack R. W. Kalbacher H. Jung G. Gotz F. (1999). Inactivation of the dlt operon in *Staphylococcus aureus* confers sensitivity to defensins, protegrins, and other antimicrobial peptides. J. Biol. Chem. 274, 8405–8410. doi: 10.1074/jbc.274.13.8405 10085071

[ref46] Peschel A. Vuong C. Otto M. Gotz F. (2000). The D-alanine residues of *Staphylococcus aureus* teichoic acids alter the susceptibility to vancomycin and the activity of autolytic enzymes. Antimicrob. Agents Chemother. 44, 2845–2847. doi: 10.1128/AAC.44.10.2845-2847.2000, PMID: 10991869 PMC90160

[ref47] Rasigade J. P. Dumitrescu O. Lina G. (2014). New epidemiology of *Staphylococcus aureus* infections. Clin. Microbiol. Infect. 20, 587–588. doi: 10.1111/1469-0691.12718, PMID: 24930666

[ref48] Santa Maria J. P. Sadaka A. Moussa S. H. Brown S. Zhang Y. J. Rubin E. J. . (2014). Compound-gene interaction mapping reveals distinct roles for *Staphylococcus aureus* teichoic acids. Proc. Natl. Acad. Sci. USA 111, 12510–12515. doi: 10.1073/pnas.1404099111, PMID: 25104751 PMC4151746

[ref49] Sato'o Y. Aiba Y. Kiga K. Watanabe S. Sasahara T. Hayakawa Y. . (2018). Optimized universal protocol for electroporation of both coagulase-positive and -negative staphylococci. J. Microbiol. Methods 146, 25–32. doi: 10.1016/j.mimet.2018.01.006, PMID: 29355575

[ref50] Scientific Committee on Consumer Bernauer U. (2015). Opinion of the scientific committee on consumer safety (SCCS)--2nd revision of the safety of the use of poly(hexamethylene) biguanide hydrochloride or polyaminopropyl biguanide (PHMB) in cosmetic products. Regul. Toxicol. Pharmacol. 73, 885–886. doi: 10.1016/j.yrtph.2015.09.035 26456666

[ref51] Sowlati-Hashjin S. Carbone P. Karttunen M. (2020). Insights into the Polyhexamethylene Biguanide (PHMB) mechanism of action on bacterial membrane and DNA: a molecular dynamics study. J. Phys. Chem. B 124, 4487–4497. doi: 10.1021/acs.jpcb.0c02609, PMID: 32390430

[ref52] Stickler D. J. (2002). Susceptibility of antibiotic-resistant gram-negative bacteria to biocides: a perspective from the study of catheter biofilms. J. Appl. Microbiol. 92, 163S–170S. doi: 10.1046/j.1365-2672.92.5s1.6.x 12000625

[ref53] Theophel K. Schacht V. J. Schluter M. Schnell S. Stingu C. S. Schaumann R. . (2014). The importance of growth kinetic analysis in determining bacterial susceptibility against antibiotics and silver nanoparticles. Front. Microbiol. 5:544. doi: 10.3389/fmicb.2014.00544 25426104 PMC4226228

[ref54] To E. Dyck R. Gerber S. Kadavil S. Woo K. Y. (2016). The effectiveness of topical Polyhexamethylene Biguanide (PHMB) agents for the treatment of chronic wounds: a systematic review. Surg. Technol. Int. 29, 45–51, PMID: 27608742

[ref55] Tong S. Y. Davis J. S. Eichenberger E. Holland T. L. Fowler V. G. Jr. (2015). *Staphylococcus aureus* infections: epidemiology, pathophysiology, clinical manifestations, and management. Clin. Microbiol. Rev. 28, 603–661. doi: 10.1128/CMR.00134-14, PMID: 26016486 PMC4451395

[ref56] Vaska V. L. Nimmo G. R. Jones M. Grimwood K. Paterson D. L. (2012). Increases in Australian cutaneous abscess hospitalisations: 1999-2008. Eur. J. Clin. Microbiol. Infect. Dis. 31, 93–96. doi: 10.1007/s10096-011-1281-3, PMID: 21553298

[ref57] Verspecht T. Rodriguez Herrero E. Khodaparast L. Khodaparast L. Boon N. Bernaerts K. . (2019). Development of antiseptic adaptation and cross-adapatation in selected oral pathogens in vitro. Sci. Rep. 9:8326. doi: 10.1038/s41598-019-44822-y, PMID: 31171824 PMC6554408

[ref58] Wertheim H. F. Melles D. C. Vos M. C. van Leeuwen W. Belkum A. Verbrugh H. A. . (2005). The role of nasal carriage in *Staphylococcus aureus* infections. Lancet Infect. Dis. 5, 751–762. doi: 10.1016/S1473-3099(05)70295-4, PMID: 16310147

[ref59] Wessels S. Ingmer H. (2013). Modes of action of three disinfectant active substances: a review. Regul. Toxicol. Pharmacol. 67, 456–467. doi: 10.1016/j.yrtph.2013.09.006, PMID: 24080225

[ref60] Wood B. M. Santa Maria J. P. Matano L. M. Vickery C. R. Walker S. (2018). A partial reconstitution implicates DltD in catalyzing lipoteichoic acid d-alanylation. J. Biol. Chem. 293, 17985–17996. doi: 10.1074/jbc.RA118.004561 30237166 PMC6240853

[ref61] Xiao Y. H. Giske C. G. Wei Z. Q. Shen P. Heddini A. Li L. J. (2011). Epidemiology and characteristics of antimicrobial resistance in China. Drug Resist. Updat. 14, 236–250. doi: 10.1016/j.drup.2011.07.001, PMID: 21807550

[ref62] Yim S. L. Cheung J. W. Cheng I. Y. Ho L. W. Szeto S. S. Chan P. . (2023). Longitudinal study on the antimicrobial performance of a Polyhexamethylene Biguanide (PHMB)-treated textile fabric in a hospital environment. Polymers (Basel) 15:1203. doi: 10.3390/polym15051203 36904444 PMC10007333

