# Supplementary material for: The dltC gene contributes to polyhexamethylene biguanide resistance in Staphylococcus aureus
Source: Front Microbiol. 2025 Nov 13;16:1681222. doi: 10.3389/fmicb.2025.1681222 (PMC12662226; doi:10.3389/fmicb.2025.1681222)
Supplement: Supplementary file 1 [file Table_1.docx]

Figure S1


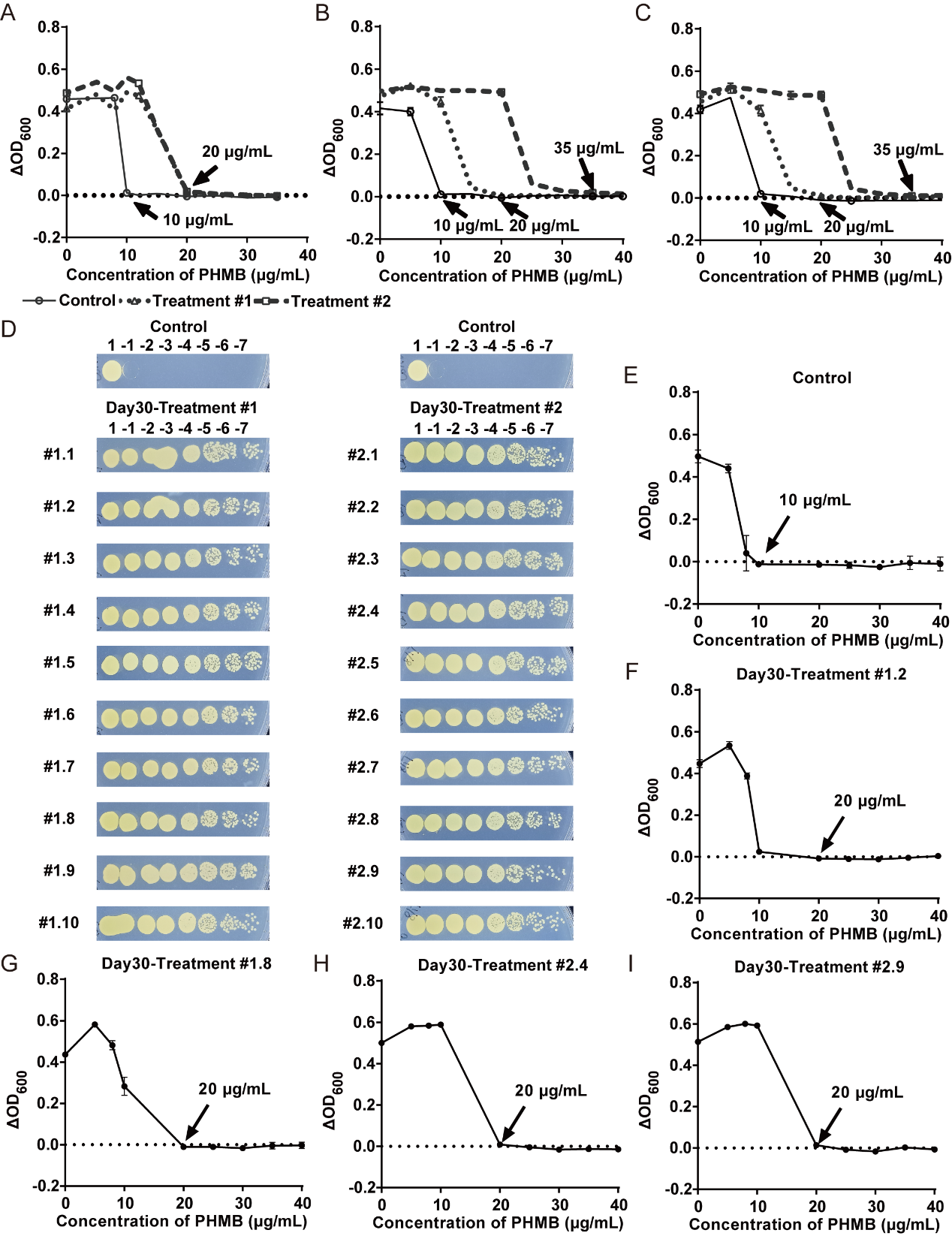


Figure S1. MIC of treatment groups gradually increased under continuous 2 μg/mL PHMB exposure. MRSA-PR #1.2, #1.8, #2.4, and #2.9 were selected for further experiments. (A-C) MIC values of control and treatment groups (#1 and #2) after 10, 20, and 30 days of PHMB exposure. (D) Spot assay of 10 monoclonal isolates from control, treatment #1, and #2 on 10 μg/mL PHMB plates. (E-I) MIC values of wild-type and selected clones (MRSA-PR #1.2, #1.8, #2.4, #2.9). Treatment groups #1 and #2 received identical protocols but showed different MIC changes during the experiment, and were both included for further analysis.

Figure S2

**
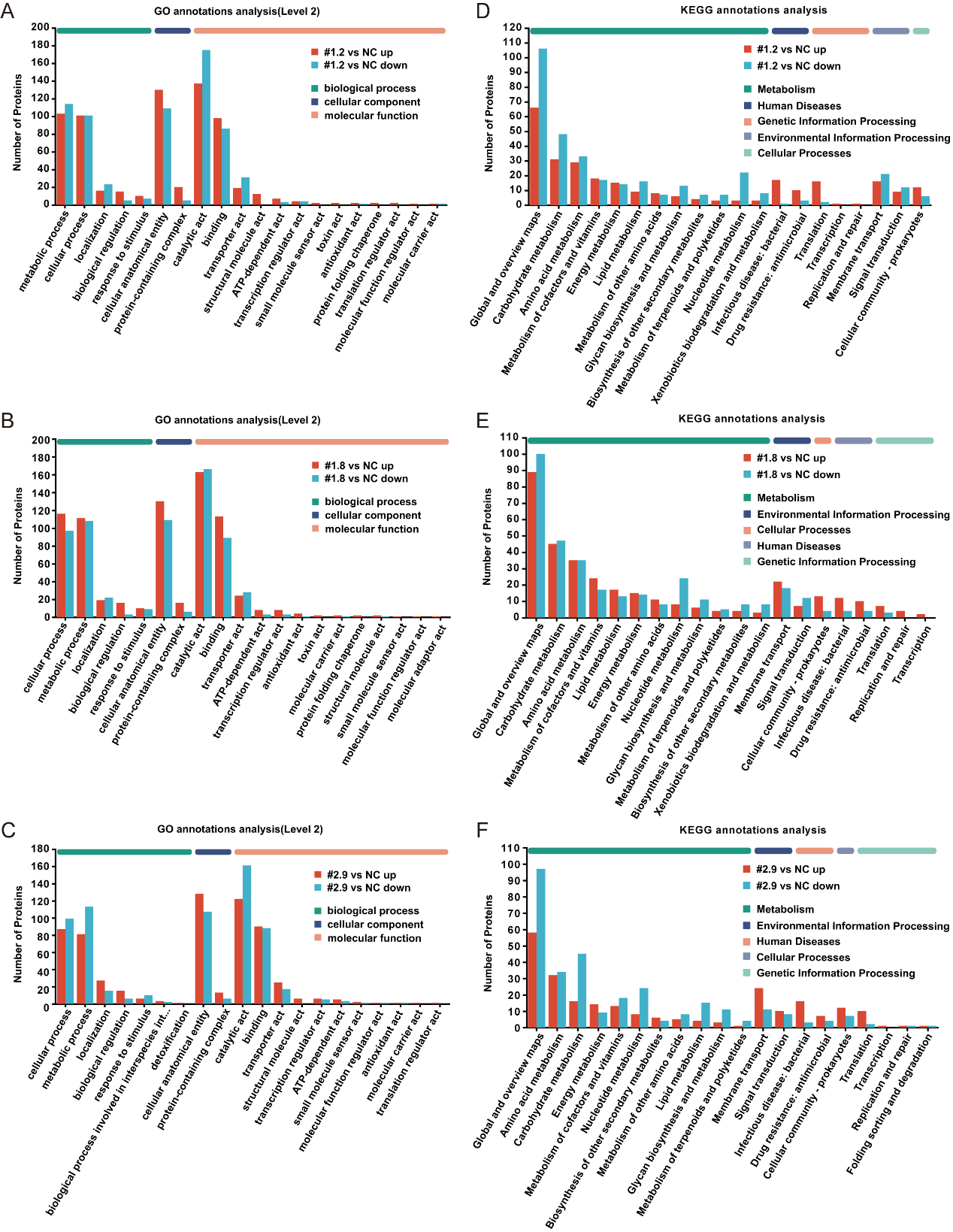
**

**Figure S2. Functional similarity analysis of differentially expressed proteins in MRSA-PR#1.2, #1.8, and #2.9.** (A) GO classification annotation of differentially expressed proteins in the three MRSA-PR strains. Functional enrichment of upregulated and downregulated proteins was analyzed separately, with the top 20 GO terms selected based on –log10(p-value). (B) KEGG pathway classification annotation of differentially expressed proteins in the three strains. Functional enrichment of upregulated and downregulated proteins was analyzed separately, with the top 20 pathways selected based on –log10(p-value).
