# Supplementary material for: The dltC gene contributes to polyhexamethylene biguanide resistance in Staphylococcus aureus
Source: Front Microbiol. 2025 Nov 13;16:1681222. doi: 10.3389/fmicb.2025.1681222 (PMC12662226; doi:10.3389/fmicb.2025.1681222)
Supplement: Supplementary file 2 [file Table_2.docx]

Table 1 Primers used in this work

| Primer name | Sequence（5’→3’） |
| --- | --- |
| Upstream 5’F-1 | TTTGAGATCTGTCCATACCCATGGTCTAGACGTCGTATGGCAAGTTTTATTGA |
| Upstream 3’R | TTAAATTCTCCTTTATTATATAAGTTTACCTGAGAAGATTAAAAAGCC |
| Downstream 5’F | TAAACTTATATAATAAAGGAGAATTTAAATGAAATTAAAACCTTTTTT |
| Downstream 3’R -1 | AAGATACAGGTATATTTTTCTGACTCGAGCCGATGTGTACGGCATCACT |
| promoter-F-*Xba*I | GTCGACTCTAGAGGATATGTGATGAGTTTATTTGAT |
| promoter-R | ATTGCACCTCTTAAAGTTCTTAGTAAAAACGC |
| *dltC*-F | GAACTTTAAGAGGTGCAATTTGGCTTTTTAATCTTCTCA |
| *dltC*-R-*EcoR*I | TCTTCAAGAATTCGAGAATACCGCTCCACTAATTAA |
| PCRVerify-F | CGAACGACCTACACCGAACT |
| PCRVerify-R | CACACATCCAGGTGGTTCAT |
| *dltC*-sgRNA-F-1 | GAAAGACGTAGAAATTTTTGAAGA |
| *dltC*-sgRNA-R-1 | AAACTCTTCAAAAATTTCTACGTC |
| pLI50-insert-F | CTAAAAACCTACAGAAGCTTGCATGCCT |
| pLI50-AmpR-F | AATGGTTTCTTAGACGTCAGGT |
| pLI50-AmpR-R | CAAAAAGGATCTTCACCTAGATCC |
| out-*dltC*-F | TGCAATGTCTAACGTGGCAT |
| in-*dltC*-F | TGATTCTTTCCAAACAGTTGGATT |
| in-*dltC*-R | TGGTGTTGCCCACTCATCTC |
| *gyrA*-F | TCCCTGAATCAACATTACGTCC |
| *gyrA*-R | CCCTACAACTTCGTCACCTTC |
